# Propane-1,2-diaminium bis­(pyridine-2,6-dicarboxyl­ato-κ^3^ *O* ^2^,*N*,*O* ^6^)mercurate(II) dihydrate

**DOI:** 10.1107/S1600536811024366

**Published:** 2011-06-25

**Authors:** Ali Akbar Agah, Hamid Reza Saadati Moshtaghin, Behrouz Notash, Hadi Amiri Rudbari, Giuseppe Bruno

**Affiliations:** a Faculty of Chemistry, Tarbiat Moallem University, 15614 Tehran, Iran; b Department of Chemistry, Shahid Beheshti University, G. C., Evin, Tehran 1983963113, Iran; c Dipartimento di Chimica Inorganica, Vill. S. Agata, Salita Sperone 31, Universita di Messina, 98166 Messina, Italy

## Abstract

In the title compound, (C_3_H_12_N_2_)[Hg(C_7_H_3_NO_4_)_2_]·2H_2_O, the Hg^II^ ion is coordinated by four O and two N atoms of two pyridine-2,6-dicarboxyl­ate (pydc) ligands in a distorted octa­hedral environment. The structure contains two uncoordinated water mol­ecules. In the crystal, N—H⋯O, O—H⋯O and weak C—H⋯O hydrogen bonds and π–π stacking inter­actions between the pyridine rings of the pydc ligands, with a centroid–centroid distance of 3.4582 (18) Å, stabilize the structure.

## Related literature

For related structures, see: Aghabozorg *et al.* (2008*a* ▶,*b* ▶,*c* ▶,*d* ▶); Pasdar *et al.* (2011 ▶).
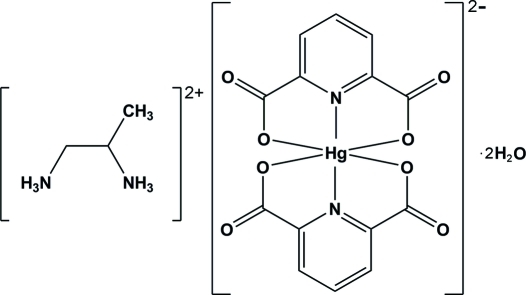


## Experimental

### 

#### Crystal data

(C_3_H_12_N_2_)[Hg(C_7_H_3_NO_4_)_2_]·2H_2_O
*M* *_r_* = 642.98
Triclinic, 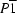

*a* = 8.627 (3) Å
*b* = 10.253 (4) Å
*c* = 13.307 (5) Å
α = 86.33 (1)°
β = 74.08 (2)°
γ = 65.18 (1)°
*V* = 1025.6 (7) Å^3^
*Z* = 2
Mo *K*α radiation
μ = 7.57 mm^−1^
*T* = 293 K
0.40 × 0.30 × 0.20 mm

#### Data collection

Bruker APEXII CCD diffractometer
Absorption correction: multi-scan (*SADABS*; Sheldrick, 1996 ▶) *T*_min_ = 0.384, *T*_max_ = 0.746
42213 measured reflections
6084 independent reflections
5880 reflections with *I* > 2σ(*I*)
*R*_int_ = 0.034

#### Refinement

*R*[*F* ^2^ > 2σ(*F* ^2^)] = 0.017
*wR*(*F* ^2^) = 0.041
*S* = 1.13
6084 reflections
306 parameters
1 restraint
H atoms treated by a mixture of independent and constrained refinement
Δρ_max_ = 0.86 e Å^−3^
Δρ_min_ = −1.28 e Å^−3^

### 

Data collection: *APEX2* (Bruker, 2007 ▶); cell refinement: *SAINT* (Bruker, 2007 ▶); data reduction: *SAINT*; program(s) used to solve structure: *SHELXS97* (Sheldrick, 2008 ▶); program(s) used to refine structure: *SHELXL97* (Sheldrick, 2008 ▶); molecular graphics: *ORTEP-3* (Farrugia, 1997 ▶); software used to prepare material for publication: *SHELXTL* (Sheldrick, 2008 ▶).

## Supplementary Material

Crystal structure: contains datablock(s) I, global. DOI: 10.1107/S1600536811024366/hy2434sup1.cif

Structure factors: contains datablock(s) I. DOI: 10.1107/S1600536811024366/hy2434Isup2.hkl

Additional supplementary materials:  crystallographic information; 3D view; checkCIF report

## Figures and Tables

**Table 1:** Hydrogen-bond geometry (Å, °)

*D*—H⋯*A*	*D*—H	H⋯*A*	*D*⋯*A*	*D*—H⋯*A*
O9—H9*A*⋯O5^i^	0.72 (5)	2.23 (5)	2.821 (3)	140 (5)
O9—H9*B*⋯O6^ii^	0.70 (6)	2.48 (7)	3.017 (4)	135 (8)
O10—H10*A*⋯O2	0.73 (4)	1.99 (4)	2.718 (3)	170 (4)
O10—H10*B*⋯O1^iii^	0.72 (4)	2.11 (4)	2.812 (3)	166 (4)
N1—H1*A*⋯O5^iv^	0.89	1.99	2.876 (3)	174
N1—H1*B*⋯O7	0.89	1.96	2.836 (2)	168
N1—H1*C*⋯O10^v^	0.89	1.94	2.806 (3)	165
N2—H2*A*⋯O9	0.89	1.93	2.802 (3)	165
N2—H2*B*⋯O4^iv^	0.89	1.96	2.845 (2)	176
N2—H2*C*⋯O1^vi^	0.89	1.96	2.809 (3)	158
C10—H10⋯O3^vii^	0.93	2.40	3.197 (3)	144
C12—H12⋯O4^i^	0.93	2.48	3.232 (3)	138

Symmetry codes: (i) 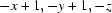; (ii) 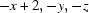; (iii) 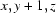; (iv) 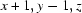; (v) 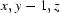; (vi) 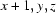; (vii) 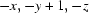.

**Fig. 1.**
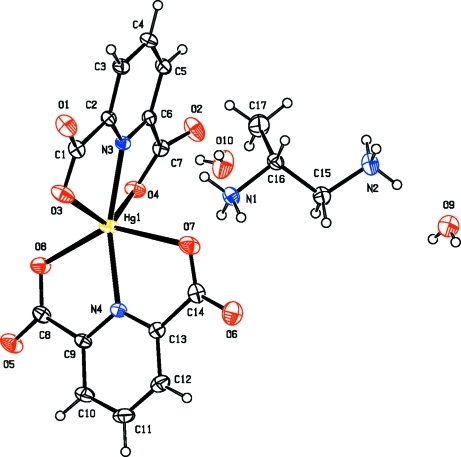
The molecular structure of the title compound, with displacement ellipsoids drawn at 30% probability level.

**Fig. 2.**
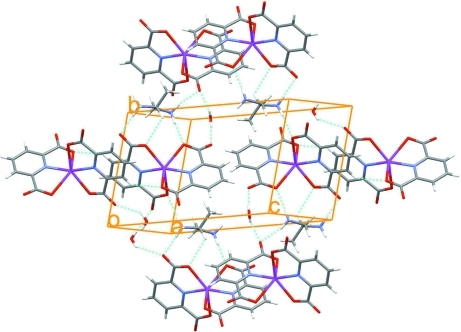
The packing diagram of the title compound. Hydrogen bonds are shown as blue dashed lines.

**Fig. 3.**
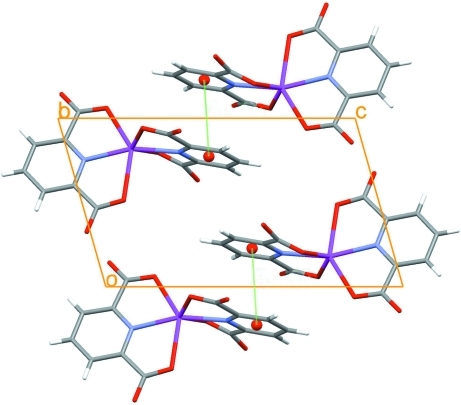
The packing diagram of the title compound viewed down the a-axis, showing π–π interactions between the pydc ligands [centroid–centroid distance = 3.4582 (18) Å]. Only anionic parts are shown for clarity.

## supplementary crystallographic information

### Comment

Our research group has previously reported several proton-transfer compounds using pyridine-2,6-dicarboxylic acid (pydcH_2_), propane-1,2-diamine (p-1,2-da) and propane-1,3-diamine (p-1,3-da), including (p-1,2-daH_2_)(pydcH)_2_.2H_2_O (Aghabozorg *et al.*, 2008*d*), (p-1,3-daH_2_)[Cd(pydc)_2_].3.5H_2_O (Aghabozorg *et al.*, 2008*b*), (p-1,2-daH_2_)[Ni(pydc)_2_].4H_2_O (Aghabozorg *et al.*, 2008*c*), (p-1,3-daH_2_)[Hg(hypydc)Cl(H_2_O)]_2_.4H_2_O (hypydcH_2_ = 4-hydroxypyridine-2,6-dicarboxylic acid) (Aghabozorg *et al.*, 2008*a*) and (p-1,2-daH_2_)[Zr(pydc)_3_].3H_2_O (Pasdar *et al.*, 2011).

The molecular structure of the title compound is shown in Fig. 1. The Hg^II^ ion is six-coordinated by two pydc ligands in a distorted octahedral environment. In the crystal structure, there are intermolecular N—H···O, O—H···O and weak C—H···O hydrogen bonds (Fig. 2, Table 1). There are also π–π stacking interactions between the pyridine rings of the pydc ligands, with a centroid–centroid distance of 3.4582 (18) Å (Fig. 2). These noncovalent interactions play an important role in the stabilization of the crystal structure.

### Experimental

A mixture of an aqueous solution (30 ml) of propane-1,2-diamine (1 mmol), pyridine-2,6-dicarboxylic acid (2 mmol) and mercury(II) nitrate (1 mmol) were stirred at room temperature. Crystals of the title compound were obtained after three weeks at room temperature.

### Refinement

H atoms of the water molecules were found in a difference Fourier map and refined isotropically with a distance restraint of O9—H9B = 0.69 (2) Å. The other H atoms were positioned geometrically and refined as riding atoms, with N—H = 0.89 (NH_3_), C—H = 0.93(aromatic CH), 0.98(aliphatic CH), 0.97 (CH_2_) and 0.96 (CH_3_) Å and with *U*_iso_(H) = 1.2(1.5 for methyl)*U*_eq_(C).

### Crystal data

**Table:**

(C_3_H_12_N_2_)[Hg(C_7_H_3_NO_4_)_2_]·2H_2_O	*Z* = 2
*M**_r_* = 642.98	*F*(000) = 624
Triclinic, *P*1	*D*_x_ = 2.082 Mg m^−^^3^
Hall symbol: -P 1	Mo *K*α radiation, λ = 0.71073 Å
*a* = 8.627 (3) Å	Cell parameters from 9853 reflections
*b* = 10.253 (4) Å	θ = 2.6–30.2°
*c* = 13.307 (5) Å	µ = 7.57 mm^−^^1^
α = 86.33 (1)°	*T* = 293 K
β = 74.08 (2)°	Block, yellow
γ = 65.18 (1)°	0.40 × 0.30 × 0.20 mm
*V* = 1025.6 (7) Å^3^	

### Data collection

**Table:**

Bruker APEXII CCD diffractometer	6084 independent reflections
Radiation source: fine-focus sealed tube	5880 reflections with *I* > 2σ(*I*)
graphite	*R*_int_ = 0.034
φ and ω scans	θ_max_ = 30.2°, θ_min_ = 2.6°
Absorption correction: multi-scan (*SADABS*; Sheldrick, 1996)	*h* = −12→12
*T*_min_ = 0.384, *T*_max_ = 0.746	*k* = −14→14
42213 measured reflections	*l* = −18→18

### Refinement

**Table:**

Refinement on *F*^2^	Secondary atom site location: difference Fourier map
Least-squares matrix: full	Hydrogen site location: inferred from neighbouring sites
*R*[*F*^2^ > 2σ(*F*^2^)] = 0.017	H atoms treated by a mixture of independent and constrained refinement
*wR*(*F*^2^) = 0.041	*w* = 1/[σ^2^(*F*_o_^2^) + (0.0168*P*)^2^ + 0.6039*P*] where *P* = (*F*_o_^2^ + 2*F*_c_^2^)/3
*S* = 1.13	(Δ/σ)_max_ = 0.002
6084 reflections	Δρ_max_ = 0.86 e Å^−^^3^
306 parameters	Δρ_min_ = −1.28 e Å^−^^3^
1 restraint	Extinction correction: *SHELXL97* (Sheldrick, 2008), Fc^*^=kFc[1+0.001xFc^2^λ^3^/sin(2θ)]^-1/4^
Primary atom site location: structure-invariant direct methods	Extinction coefficient: 0.0127 (3)

### Fractional atomic coordinates and isotropic or equivalent isotropic displacement parameters (Å^2^)

**Table:**

	*x*	*y*	*z*	*U*_iso_*/*U*_eq_	
C1	0.0952 (3)	0.2606 (2)	0.35723 (16)	0.0295 (3)	
C2	0.1682 (2)	0.33158 (18)	0.41789 (14)	0.0241 (3)	
C3	0.1950 (3)	0.2868 (2)	0.51469 (15)	0.0301 (4)	
H3	0.1721	0.2096	0.5436	0.036*	
C4	0.2562 (3)	0.3584 (2)	0.56779 (15)	0.0326 (4)	
H4	0.2756	0.3296	0.6326	0.039*	
C5	0.2882 (2)	0.4740 (2)	0.52308 (15)	0.0303 (4)	
H5	0.3300	0.5232	0.5573	0.036*	
C6	0.2570 (2)	0.51473 (18)	0.42708 (14)	0.0238 (3)	
C7	0.2786 (2)	0.6458 (2)	0.37671 (15)	0.0284 (3)	
C8	−0.0385 (3)	0.6975 (2)	0.07655 (16)	0.0310 (4)	
C9	0.1281 (2)	0.58638 (19)	0.00393 (14)	0.0254 (3)	
C10	0.1569 (3)	0.5844 (2)	−0.10336 (16)	0.0336 (4)	
H10	0.0734	0.6516	−0.1338	0.040*	
C11	0.3121 (3)	0.4807 (3)	−0.16443 (17)	0.0417 (5)	
H11	0.3338	0.4768	−0.2368	0.050*	
C12	0.4349 (3)	0.3832 (3)	−0.11813 (17)	0.0377 (4)	
H12	0.5402	0.3135	−0.1589	0.045*	
C13	0.3999 (2)	0.38984 (19)	−0.01001 (15)	0.0269 (3)	
C14	0.5322 (3)	0.2844 (2)	0.04488 (17)	0.0327 (4)	
C15	0.9453 (3)	0.0165 (2)	0.18507 (18)	0.0342 (4)	
H15A	0.9845	−0.0488	0.1245	0.041*	
H15B	0.9118	0.1136	0.1615	0.041*	
C16	0.7860 (3)	0.0057 (2)	0.26298 (16)	0.0301 (3)	
H16	0.8265	−0.0875	0.2945	0.036*	
C17	0.6924 (4)	0.1229 (4)	0.3497 (2)	0.0569 (7)	
H17A	0.5935	0.1098	0.3959	0.085*	
H17B	0.6506	0.2150	0.3201	0.085*	
H17C	0.7736	0.1183	0.3882	0.085*	
N1	0.6591 (2)	0.01061 (18)	0.20471 (15)	0.0329 (3)	
H1A	0.7145	−0.0584	0.1535	0.049*	
H1B	0.6181	0.0960	0.1775	0.049*	
H1C	0.5690	−0.0029	0.2483	0.049*	
N2	1.0926 (2)	−0.0202 (2)	0.23407 (17)	0.0392 (4)	
H2A	1.1850	−0.0134	0.1877	0.059*	
H2B	1.1243	−0.1098	0.2548	0.059*	
H2C	1.0569	0.0405	0.2891	0.059*	
N3	0.19989 (19)	0.44291 (15)	0.37622 (11)	0.0230 (3)	
N4	0.2479 (2)	0.48991 (15)	0.04832 (12)	0.0237 (3)	
O1	0.0755 (2)	0.15236 (17)	0.39594 (15)	0.0441 (4)	
O2	0.3667 (3)	0.69114 (19)	0.41085 (17)	0.0491 (4)	
O3	0.0587 (3)	0.31498 (18)	0.27586 (13)	0.0433 (4)	
O4	0.2038 (2)	0.69576 (15)	0.30516 (12)	0.0347 (3)	
O5	−0.1423 (2)	0.79421 (18)	0.03529 (14)	0.0483 (4)	
O6	0.6731 (2)	0.1974 (2)	−0.01150 (17)	0.0553 (5)	
O7	0.4868 (2)	0.29338 (18)	0.14278 (13)	0.0434 (4)	
O8	−0.0605 (2)	0.68596 (16)	0.17330 (12)	0.0382 (3)	
O10	0.4210 (3)	0.9319 (3)	0.3570 (2)	0.0615 (6)	
Hg1	0.179763 (10)	0.496442 (7)	0.218696 (5)	0.03198 (4)	
O9	1.3514 (3)	0.0549 (4)	0.09976 (19)	0.0606 (5)	
H10A	0.419 (5)	0.862 (4)	0.370 (3)	0.069 (12)*	
H10B	0.334 (5)	0.989 (4)	0.377 (3)	0.052 (10)*	
H9A	1.293 (6)	0.122 (5)	0.084 (4)	0.088 (17)*	
H9B	1.393 (9)	−0.001 (6)	0.061 (5)	0.18 (3)*	

### Atomic displacement parameters (Å^2^)

**Table:**

	*U*^11^	*U*^22^	*U*^33^	*U*^12^	*U*^13^	*U*^23^
C1	0.0315 (8)	0.0228 (8)	0.0363 (9)	−0.0110 (7)	−0.0132 (7)	0.0022 (7)
C2	0.0247 (7)	0.0204 (7)	0.0261 (8)	−0.0076 (6)	−0.0089 (6)	0.0038 (6)
C3	0.0323 (9)	0.0291 (9)	0.0301 (9)	−0.0131 (7)	−0.0124 (7)	0.0114 (7)
C4	0.0351 (9)	0.0380 (10)	0.0260 (8)	−0.0133 (8)	−0.0151 (7)	0.0099 (7)
C5	0.0286 (8)	0.0347 (9)	0.0284 (9)	−0.0111 (7)	−0.0125 (7)	0.0017 (7)
C6	0.0223 (7)	0.0220 (7)	0.0260 (8)	−0.0076 (6)	−0.0078 (6)	0.0019 (6)
C7	0.0290 (8)	0.0246 (8)	0.0313 (9)	−0.0116 (7)	−0.0077 (7)	0.0030 (7)
C8	0.0365 (9)	0.0218 (8)	0.0345 (9)	−0.0084 (7)	−0.0163 (8)	0.0045 (7)
C9	0.0342 (8)	0.0220 (7)	0.0258 (8)	−0.0144 (7)	−0.0137 (7)	0.0063 (6)
C10	0.0464 (11)	0.0361 (10)	0.0287 (9)	−0.0232 (9)	−0.0186 (8)	0.0111 (7)
C11	0.0541 (13)	0.0533 (13)	0.0222 (8)	−0.0278 (11)	−0.0091 (8)	0.0034 (8)
C12	0.0392 (10)	0.0416 (11)	0.0283 (9)	−0.0173 (9)	−0.0014 (8)	−0.0032 (8)
C13	0.0298 (8)	0.0242 (8)	0.0285 (8)	−0.0134 (7)	−0.0072 (6)	0.0007 (6)
C14	0.0327 (9)	0.0227 (8)	0.0429 (11)	−0.0102 (7)	−0.0127 (8)	0.0013 (7)
C15	0.0349 (9)	0.0306 (9)	0.0407 (10)	−0.0160 (8)	−0.0138 (8)	0.0100 (8)
C16	0.0316 (8)	0.0293 (9)	0.0314 (9)	−0.0125 (7)	−0.0130 (7)	0.0060 (7)
C17	0.0466 (13)	0.0685 (18)	0.0495 (14)	−0.0182 (13)	−0.0080 (11)	−0.0214 (13)
N1	0.0321 (8)	0.0284 (8)	0.0394 (9)	−0.0107 (6)	−0.0157 (7)	0.0054 (7)
N2	0.0351 (9)	0.0301 (8)	0.0578 (12)	−0.0163 (7)	−0.0186 (8)	0.0111 (8)
N3	0.0256 (6)	0.0198 (6)	0.0219 (6)	−0.0070 (5)	−0.0082 (5)	0.0028 (5)
N4	0.0307 (7)	0.0191 (6)	0.0234 (6)	−0.0109 (6)	−0.0102 (5)	0.0033 (5)
O1	0.0553 (10)	0.0321 (8)	0.0602 (11)	−0.0265 (7)	−0.0287 (8)	0.0140 (7)
O2	0.0566 (10)	0.0410 (9)	0.0735 (12)	−0.0324 (8)	−0.0384 (9)	0.0194 (8)
O3	0.0666 (11)	0.0395 (8)	0.0414 (8)	−0.0293 (8)	−0.0320 (8)	0.0098 (7)
O4	0.0499 (8)	0.0271 (7)	0.0328 (7)	−0.0192 (6)	−0.0166 (6)	0.0087 (5)
O5	0.0521 (9)	0.0334 (8)	0.0470 (9)	0.0010 (7)	−0.0262 (8)	0.0068 (7)
O6	0.0390 (9)	0.0432 (10)	0.0602 (12)	0.0033 (7)	−0.0084 (8)	−0.0071 (8)
O7	0.0461 (9)	0.0327 (8)	0.0399 (8)	−0.0019 (6)	−0.0187 (7)	0.0054 (6)
O8	0.0409 (8)	0.0298 (7)	0.0304 (7)	−0.0017 (6)	−0.0104 (6)	0.0010 (6)
O10	0.0463 (11)	0.0377 (10)	0.0917 (17)	−0.0187 (9)	−0.0075 (11)	0.0200 (11)
Hg1	0.04592 (5)	0.02638 (5)	0.02169 (4)	−0.01097 (3)	−0.01378 (3)	0.00471 (2)
O9	0.0505 (11)	0.0801 (17)	0.0505 (12)	−0.0271 (12)	−0.0126 (9)	0.0001 (12)

### Geometric parameters (Å, °)

**Table:**

C1—O3	1.243 (2)	C14—O6	1.230 (3)
C1—O1	1.250 (2)	C14—O7	1.251 (3)
C1—C2	1.522 (2)	C15—N2	1.484 (3)
C2—N3	1.335 (2)	C15—C16	1.519 (3)
C2—C3	1.386 (2)	C15—H15A	0.9700
C3—C4	1.383 (3)	C15—H15B	0.9700
C3—H3	0.9300	C16—N1	1.488 (2)
C4—C5	1.389 (3)	C16—C17	1.509 (3)
C4—H4	0.9300	C16—H16	0.9800
C5—C6	1.380 (3)	C17—H17A	0.9600
C5—H5	0.9300	C17—H17B	0.9600
C6—N3	1.339 (2)	C17—H17C	0.9600
C6—C7	1.522 (2)	N1—H1A	0.8900
C7—O2	1.231 (2)	N1—H1B	0.8900
C7—O4	1.258 (2)	N1—H1C	0.8900
C8—O5	1.242 (2)	N2—H2A	0.8900
C8—O8	1.254 (2)	N2—H2B	0.8900
C8—C9	1.517 (3)	N2—H2C	0.8900
C9—N4	1.340 (2)	N3—Hg1	2.1674 (14)
C9—C10	1.380 (3)	N4—Hg1	2.1783 (15)
C10—C11	1.379 (3)	O3—Hg1	2.4786 (16)
C10—H10	0.9300	O4—Hg1	2.5159 (14)
C11—C12	1.375 (3)	O7—Hg1	2.5577 (16)
C11—H11	0.9300	O8—Hg1	2.3647 (15)
C12—C13	1.387 (3)	O10—H10A	0.73 (4)
C12—H12	0.9300	O10—H10B	0.72 (4)
C13—N4	1.338 (2)	O9—H9A	0.72 (5)
C13—C14	1.524 (3)	O9—H9B	0.69 (2)
			
O3—C1—O1	126.09 (18)	C17—C16—C15	113.74 (19)
O3—C1—C2	118.19 (16)	N1—C16—H16	108.4
O1—C1—C2	115.71 (17)	C17—C16—H16	108.4
N3—C2—C3	120.68 (16)	C15—C16—H16	108.4
N3—C2—C1	117.50 (15)	C16—C17—H17A	109.5
C3—C2—C1	121.77 (16)	C16—C17—H17B	109.5
C4—C3—C2	119.22 (17)	H17A—C17—H17B	109.5
C4—C3—H3	120.4	C16—C17—H17C	109.5
C2—C3—H3	120.4	H17A—C17—H17C	109.5
C3—C4—C5	119.17 (17)	H17B—C17—H17C	109.5
C3—C4—H4	120.4	C16—N1—H1A	109.5
C5—C4—H4	120.4	C16—N1—H1B	109.5
C6—C5—C4	118.98 (17)	H1A—N1—H1B	109.5
C6—C5—H5	120.5	C16—N1—H1C	109.5
C4—C5—H5	120.5	H1A—N1—H1C	109.5
N3—C6—C5	120.99 (16)	H1B—N1—H1C	109.5
N3—C6—C7	117.26 (15)	C15—N2—H2A	109.5
C5—C6—C7	121.70 (16)	C15—N2—H2B	109.5
O2—C7—O4	127.38 (18)	H2A—N2—H2B	109.5
O2—C7—C6	116.32 (17)	C15—N2—H2C	109.5
O4—C7—C6	116.30 (16)	H2A—N2—H2C	109.5
O5—C8—O8	124.9 (2)	H2B—N2—H2C	109.5
O5—C8—C9	117.18 (18)	C2—N3—C6	120.94 (15)
O8—C8—C9	117.94 (16)	C2—N3—Hg1	119.33 (12)
N4—C9—C10	121.04 (18)	C6—N3—Hg1	119.53 (12)
N4—C9—C8	117.12 (16)	C13—N4—C9	121.05 (16)
C10—C9—C8	121.83 (17)	C13—N4—Hg1	121.45 (12)
C11—C10—C9	118.54 (19)	C9—N4—Hg1	117.44 (12)
C11—C10—H10	120.7	C1—O3—Hg1	111.54 (12)
C9—C10—H10	120.7	C7—O4—Hg1	109.10 (12)
C12—C11—C10	119.94 (19)	C14—O7—Hg1	112.51 (13)
C12—C11—H11	120.0	C8—O8—Hg1	113.91 (13)
C10—C11—H11	120.0	H10A—O10—H10B	110 (4)
C11—C12—C13	119.3 (2)	N3—Hg1—N4	160.59 (6)
C11—C12—H12	120.4	N3—Hg1—O8	126.00 (5)
C13—C12—H12	120.4	N4—Hg1—O8	73.40 (5)
N4—C13—C12	120.12 (18)	N3—Hg1—O3	71.57 (5)
N4—C13—C14	118.65 (17)	N4—Hg1—O3	107.28 (5)
C12—C13—C14	121.23 (18)	O8—Hg1—O3	102.19 (6)
O6—C14—O7	126.1 (2)	N3—Hg1—O4	70.34 (5)
O6—C14—C13	116.7 (2)	N4—Hg1—O4	115.63 (5)
O7—C14—C13	117.17 (17)	O8—Hg1—O4	84.08 (5)
N2—C15—C16	110.37 (17)	O3—Hg1—O4	136.51 (5)
N2—C15—H15A	109.6	N3—Hg1—O7	90.47 (5)
C16—C15—H15A	109.6	N4—Hg1—O7	70.12 (5)
N2—C15—H15B	109.6	O8—Hg1—O7	143.51 (5)
C16—C15—H15B	109.6	O3—Hg1—O7	89.59 (6)
H15A—C15—H15B	108.1	O4—Hg1—O7	110.80 (6)
N1—C16—C17	109.51 (18)	H9A—O9—H9B	114 (7)
N1—C16—C15	108.21 (16)		

### Hydrogen-bond geometry (Å, °)

**Table:**

*D*—H···*A*	*D*—H	H···*A*	*D*···*A*	*D*—H···*A*
O9—H9A···O5^i^	0.72 (5)	2.23 (5)	2.821 (3)	140 (5)
O9—H9B···O6^ii^	0.70 (6)	2.48 (7)	3.017 (4)	135 (8)
O10—H10A···O2	0.73 (4)	1.99 (4)	2.718 (3)	170 (4)
O10—H10B···O1^iii^	0.72 (4)	2.11 (4)	2.812 (3)	166 (4)
N1—H1A···O5^iv^	0.89	1.99	2.876 (3)	174
N1—H1B···O7	0.89	1.96	2.836 (2)	168
N1—H1C···O10^v^	0.89	1.94	2.806 (3)	165
N2—H2A···O9	0.89	1.93	2.802 (3)	165
N2—H2B···O4^iv^	0.89	1.96	2.845 (2)	176
N2—H2C···O1^vi^	0.89	1.96	2.809 (3)	158
C10—H10···O3^vii^	0.93	2.40	3.197 (3)	144
C12—H12···O4^i^	0.93	2.48	3.232 (3)	138

Symmetry codes: (i) −*x*+1, −*y*+1, −*z*; (ii) −*x*+2, −*y*, −*z*; (iii) *x*, *y*+1, *z*; (iv) *x*+1, *y*−1, *z*; (v) *x*, *y*−1, *z*; (vi) *x*+1, *y*, *z*; (vii) −*x*, −*y*+1, −*z*.
